# Tick-borne pathogens in Swedish wild boar and their attached ticks: the first detection of *Borrelia* species in wild boar in northern Europe

**DOI:** 10.1186/s13028-026-00898-x

**Published:** 2026-11-10

**Authors:** Hanna Lindell, Kari Lehtilä, Patrik Dinnétz

**Affiliations:** Department of Natural Sciences, Technology and Environmental Studies, Södertörn University, Alfred Nobels Allé 7, Huddinge, SE-141 52 Sweden

**Keywords:** *Anaplasma phagocytophilum*, Ecotyping, Feeding duration, *Ixodes ricinus*, Quantitative PCR, *Sus scrofa*, Wildlife disease ecology

## Abstract

**Background:**

The wild boar (*Sus scrofa*) population has expanded in northern Europe, yet their role in the ecology of tick-borne pathogens remains poorly understood. This study investigated the occurrence of *Anaplasma phagocytophilum* and *Borrelia burgdorferi* sensu lato in 88 Swedish wild boar and their attached *Ixodes ricinus* ticks by analysing multiple tissues (spleen, heart, urinary bladder and skin) using quantitative PCR.

**Results:**

*Anaplasma phagocytophilum* was detected in 20 (22.7%) wild boar, with infections (i.e. quantitative PCR positive) more common in juveniles than adults. Infection probability was lowest in spring but markedly higher in summer and autumn. *Borrelia* species DNA was detected in three juvenile males (3.4%), representing the first report of *Borrelia* species in wild boar in northern Europe. Thirty attached ticks were collected from 17 wild boar. Six ticks carried *A. phagocytophilum*, five carried *Borrelia* species, and one was coinfected. Ticks were more likely to be infected when collected from infected wild boar than from uninfected hosts, while feeding duration showed no detectable association with infection in either hosts or ticks.

**Conclusions:**

These findings provide new insight into the occurrence of tick-borne pathogens in wild boar and highlight the value of multi-tissue sampling and paired host–tick data for understanding pathogen dynamics in wildlife populations.

## Background

Tick-borne pathogens such as *Borrelia burgdorferi* sensu lato (s.l.) and *Anaplasma phagocytophilum* are widely distributed across Europe and maintained through enzootic transmission cycles involving *Ixodes* ticks and a diverse range of vertebrate hosts. Although the roles of small mammals and birds as reservoir hosts are well established, and large mammals are recognised as important hosts supporting tick feeding and reproduction, the contribution of many wildlife species to pathogen maintenance and transmission remains poorly understood [1–3].

Wild boar (*Sus scrofa*) populations have expanded markedly across Europe in recent decades. In Sweden, the species had been extinct for centuries before re-establishing itself during the 1970s and 1980s following escapes from enclosures. Since then, the wild boar population has grown rapidly, increasing almost exponentially until around 2010–2011, when growth began to level off. Today, wild boar are widely distributed across southern and central Sweden, with occasional observations further north along the coast. The total Swedish population is estimated to approximately 300,000 individuals [4–6]. This substantial increase in abundance and geographical range, together with their frequent occurrence in human-dominated landscapes highlights the importance of understanding the role of wild boar in the eco-epidemiology of tick-borne diseases.

Wild boar have been found to harbour several tick-borne pathogens, including *Anaplasma phagocytophilum*, *Borrelia* spp., *Babesia* spp., and *Neoehrlichia mikurensis* [7–10]. Among these, *A. phagocytophilum* and *Borrelia burgdorferi* s.l. are the most commonly reported tick-borne pathogens affecting humans and domestic animals in Sweden, making the role of wild boar in their maintenance and transmission a particularly relevant topic of investigation [11, 12]. Both pathogens are primarily transmitted by *Ixodes ricinus*, the most widespread and abundant tick species in Sweden. As a host generalist, *I. ricinus* feeds on a wide range of vertebrate hosts, allowing it to acquire and transmit pathogens across different host species. Other tick species also occur in Sweden, but to our knowledge, all of them have more limited distributions or are more closely associated with particular host groups, such as birds or bats, making them less likely to contribute substantially to the transmission of *A. phagocytophilum* and *B. burgdorferi* s.l in Swedish ecosystems [13, 14].

Both *A. phagocytophilum* and *B. burgdorferi* s.l. show considerable genetic diversity, with variants differing in host associations and epidemiological relevance. *Anaplasma phagocytophilum* is genetically divided into four ecotypes (I-IV), which are defined by distinct genetic variants associated with specific ecological niches. The ecotypes differ in host range and zoonotic potential, and in Europe all reported human cases of granulocytic anaplasmosis have been linked to ecotype I [15]. Therefore, determining which ecotypes circulate in wild boar provides more meaningful information than prevalence estimates alone, as it allows assessment of the potential zoonotic relevance of the detected infections. The *B. burgdorferi* s.l. complex consists of more than 20 genospecies, of which only a subset is known to cause human disease. In Europe, most cases of Lyme borreliosis are associated with *B. afzelii*, *B. garinii*, and *B. burgdorferi* sensu stricto. While *B. afzelii* and *B. garinii* are widespread across Europe, *B. burgdorferi* s.s. occurs less frequently and has a more restricted geographic distribution. Different genospecies differ in their host associations, tissue tropisms, and pathogenic potential. Therefore, species identification is important when assessing the eco-epidemiological role of wildlife hosts [16–19].

Understanding the role of wild boar in tick-borne disease systems also requires consideration of host-vector interactions. One important component of these interactions is tick feeding duration, as both *B. burgdorferi* s.l. and *A. phagocytophilum* generally require prolonged attachment for successful transmission [20, 21]. Estimates of feeding duration are used in human medicine to assess the risk of tick-borne pathogen transmission and to guide decisions regarding post-exposure prophylaxis [22, 23]. However, comparable estimates from free-ranging wildlife are rare. To our knowledge, estimates of tick feeding duration under natural conditions have so far only been reported for roe deer [24], making this the first study to investigate tick feeding duration in wild boar. Previous investigations have also typically focused on a single host tissue most commonly blood or spleen [7, 25–29], or examined either hosts or ticks in isolation, restricting opportunities to compare infection (i.e. quantitative PCR positive) status across different sample types collected from the same host individual. Low and uneven bacterial loads of both *Borrelia* spp. and *A. phagocytophilum* further complicate detection when only one tissue type is sampled. Together, these factors make it difficult to assess how often infections coincide in hosts and their attached ticks, and how feeding duration may affect these infection patterns.

A few earlier studies have investigated wild boar as potential hosts of *Borrelia* spp. and *A. phagocytophilum*, revealing marked geographic variation in prevalence across Europe [7, 9, 25, 26, 30], but data from northern Europe remain very scarce. The aim of this study is to investigate the occurrence and genetic variation of *B. burgdorferi* s.l. and *A. phagocytophilum* in wild boar in Sweden by analysing multiple host tissue types in relation to infection status and feeding duration of attached *I. ricinus*. By combining host infection data with pathogen detections and feeding duration estimates from attached ticks, the study seeks to provide a more comprehensive understanding of pathogen occurrence in wild boar and their ticks in a northern European context.

## Methods

### Study area and sample collection

In February 2023, local hunting associations across Sweden were invited to participate in a study on tick-borne pathogens in wild boar. A total of 229 hunting associations were contacted, ranging from southern Sweden (Skåne) to Dalarna, which represents the present northern distribution limit of wild boar in Sweden. Of these, 32 agreed to participate. Between March 2023 and March 2025, hunters collected samples from wild boar harvested during regular hunting activities. In Sweden, adult wild boar may be hunted from 1 April to 31 January, yearlings may be hunted year-round, and sows accompanied by striped or brown piglets are protected throughout the year [31]. Because yearlings remain lawful quarry during the closed season for adults (February–March), samples could be collected across all seasons.

This study uses the same wild boar material as Lindell et al. [unpubl.], in which spleen tissue was analysed for the presence of *Neoehrlichia mikurensis*. In the present study, we extended this approach by including multiple host tissues and attached ticks to investigate the prevalence of *Borrelia spp.* and *A. phagocytophilum.*

Hunters were asked to submit approximately 1 × 1 cm tissue samples of spleen, heart, and urinary bladder from each harvested animal. When present, attached ticks and a small piece of skin from the tick attachment site were also collected. All samples were preserved in 70% ethanol and shipped to the laboratory by regular mail. Transport times typically ranged from 2 to 7 days. Upon arrival, samples were stored at − 80 °C until further processing.

**Fig. 1:**
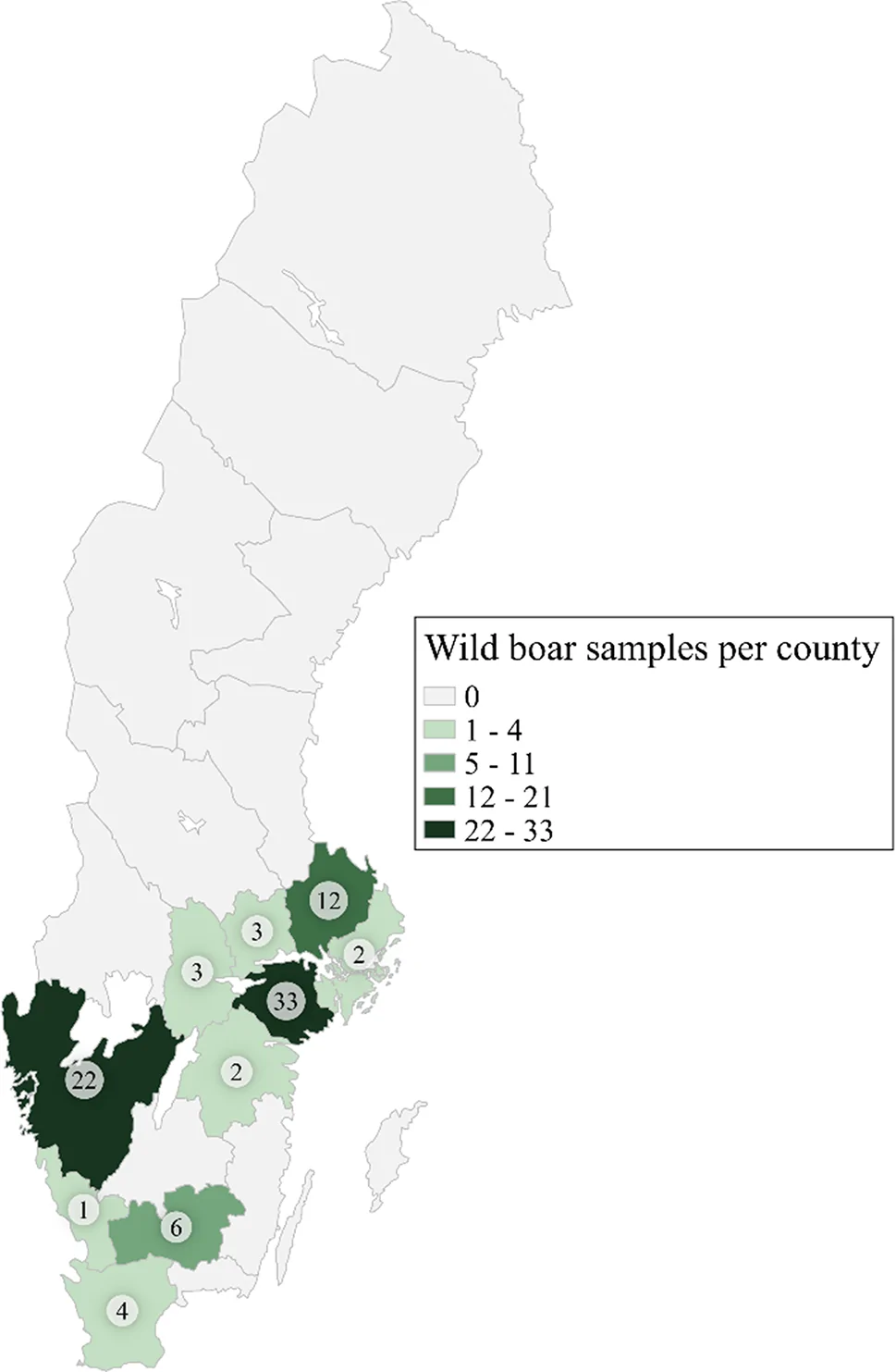
Geographical distribution of sampled wild boar across Swedish counties, 2023–2025. Darker shading indicates counties with a higher number of submitted animals. Numbers represent the sample size for each county

Wild boar were sampled across a broad geographical range in Sweden, covering ten counties: Halland (*n* = 1), Kronoberg (*n* = 6), Örebro (*n* = 3), Östergötland (*n* = 2), Skåne (*n* = 4), Södermanland (*n* = 33), Stockholm (*n* = 2), Uppsala (*n* = 12), Västmanland (*n* = 3), and Västra Götaland (*n* = 22) (Fig. 1). Several of the counties represented in this study, such as Västra Götaland, Skåne, Södermanland, and Kronoberg, are among the top-ranking counties for wild boar-related traffic accidents, a metric commonly used as an indicator of wild boar abundance [32].

Ethical approval was not required for this study because no live animals were handled or culled specifically for research purposes. All wild boars were legally harvested for human consumption in accordance with the Swedish Hunting Act (SFS 1987:259).

### DNA extraction

DNA was extracted from wild boar tissues and ticks using two different Qiagen kits. Tissue samples (spleen, heart, urinary bladder, and skin) were processed with the QIAamp Fast DNA Tissue Kit (Qiagen, Hilden, Germany) according to the manufacturer’s instructions for animal tissues. Tick DNA was extracted using the DNeasy Blood & Tissue Kit (Qiagen, Hilden, Germany), following the manufacturer’s supplementary protocol *“Purification of total DNA from ticks using the DNeasy Blood & Tissue Kit for detection of Borrelia DNA”*. All DNA extracts were stored at − 80 °C until qPCR analysis.

### qPCR analysis

DNA extracts from both wild boar tissues and ticks were analysed using TaqMan qPCR assays targeting the *gltA* gene of *A. phagocytophilum* and the 16 S rRNA gene of *B. burgdorferi* s.l. Primer and probe sequences used in both tissue and tick assays are listed in Table 1. The positive control for *A. phagocytophilum* was a plasmid containing the *A. phagocytophilum* gltA sequence (GenBank: U5972). The positive control for *Borrelia* spp. was cultured *Borrelia garinii* DNA provided by Jönköping county hospital Ryhof. The negative control was nuclease-free water.

**Table 1:** Primers and probes used in the qPCR assays

Target	Primer/probe	Sequence [5′–3′]
*A phagocytophilum*	Forward	TTTTGGGCGCTGAATACGAT
	Reverse	TCTCGAGGGAATGATCTAATAACGT
	Probe	FAM-TGCCTGAACAAGTTATG-MGB
*Borrelia* spp.	Forward	GCTGAGTCACGAAAGCGTAG
	Reverse	CACTTAACACGTTAGCTTCGGTA
	Probe	FAM-CGCTGTAAACGATGCACACTTGGT-MGB

Tissue extracts (spleen, heart, urinary bladder, and skin) were analysed with a TaqMan Exogenous Internal Positive Control (IPC, Applied Biosystems, Foster City, CA, USA) to monitor potential PCR inhibition. Tissue reactions (50 µL) consisted of 25 µL TaqPath™ BactoPure™ Microbial Detection Master Mix, 5 µL 10× IPC Mix, 1 µL 50× IPC DNA, 2.5 µL of each primer, 1 µL probe, 8 µL nuclease-free water, and 5 µL template DNA. Cycling conditions for tissue samples were 50 °C for 2 min, 95 °C for 10 min followed by 55 cycles of 95 °C for 15 s and 60 °C for 1 min. A tissue sample was considered positive when a clear amplification curve with a Cq < 46 was obtained. Only one tissue sample, a urinary bladder sample analysed for *Borrelia spp.*, showed signs of PCR inhibition, as indicated by the internal positive control. The inhibition was resolved after a 1:5 dilution of the DNA extract, allowing successful amplification.

For *A. phagocytophilum* detection in tick-DNA extracts, 25 µL reactions were prepared containing: 12.5 µL Maxima^®^ Probe qPCR Master Mix (Thermo Fisher Scientific, Waltham, MA, USA), 1.5 µL of each primer, 0.375 µL probe, 7.125 µL nuclease-free water, and 2 µL template DNA. For *Borrelia* spp., 20 µL reactions contained 10 µL Maxima^®^ Probe Master Mix, 0.4 µL of each primer, 0.4 µL probe, 6.8 µL water, and 2 µL template DNA. Cycling conditions for *A. phagocytophilum* were 95 °C for 10 min followed by 50 cycles of 95 °C for 15 s and 60 °C for 1 min. For *Borrelia* spp., reactions were initiated at 95 °C for 5 min and followed by 50 cycles of 95 °C for 10 s and 60 °C for 60 s. Tick sample was considered positive when a clear amplification curve with Cq < 40 was obtained.

### Ecotyping of *Anaplasma phagocytophilum* and species identification of *Borrelia* spp

All samples that tested positive for *Anaplasma phagocytophilum* by qPCR were subsequently analysed for ecotype assignment. A fragment of the *groEL* gene of *A. phagocytophilum* was amplified using a heminested PCR strategy previously described [33]. Positive PCR products were sequenced bidirectionally (Microsynth AG, Balgach, Switzerland). Obtained *groEL* sequences were analysed together with reference sequences representing the four *A. phagocytophilum* ecotypes as previously described [15]. Phylogenetic trees were constructed using PhyML, implemented via the NGPhylogeny.fr web platform [34], using the maximum likelihood model. Node support was assessed by non-parametric bootstrap analysis with 100 replicates and samples were assigned to ecotypes based on their clustering with reference sequences of known ecotype identity.

Species determination of *Borrelia burgdorferi* s.l. was performed by sequencing (Microsynth AG, Balgach, Switzerland) of the 5–23 S rRNA intergenic spacer (IGS) region following nested PCR as previously described [35]. Species assignments were based on both BLAST analysis and phylogenetic comparison with representative reference sequences retrieved from GenBank, including the reference sequences previously described [35]. Phylogenetic trees were constructed using the neighbour-joining method with the Kimura 2-parameter model, and node support was assessed by bootstrap analysis with 500 replicates in MEGA 12.1 [36].

### Feeding duration

**Fig. 2:**
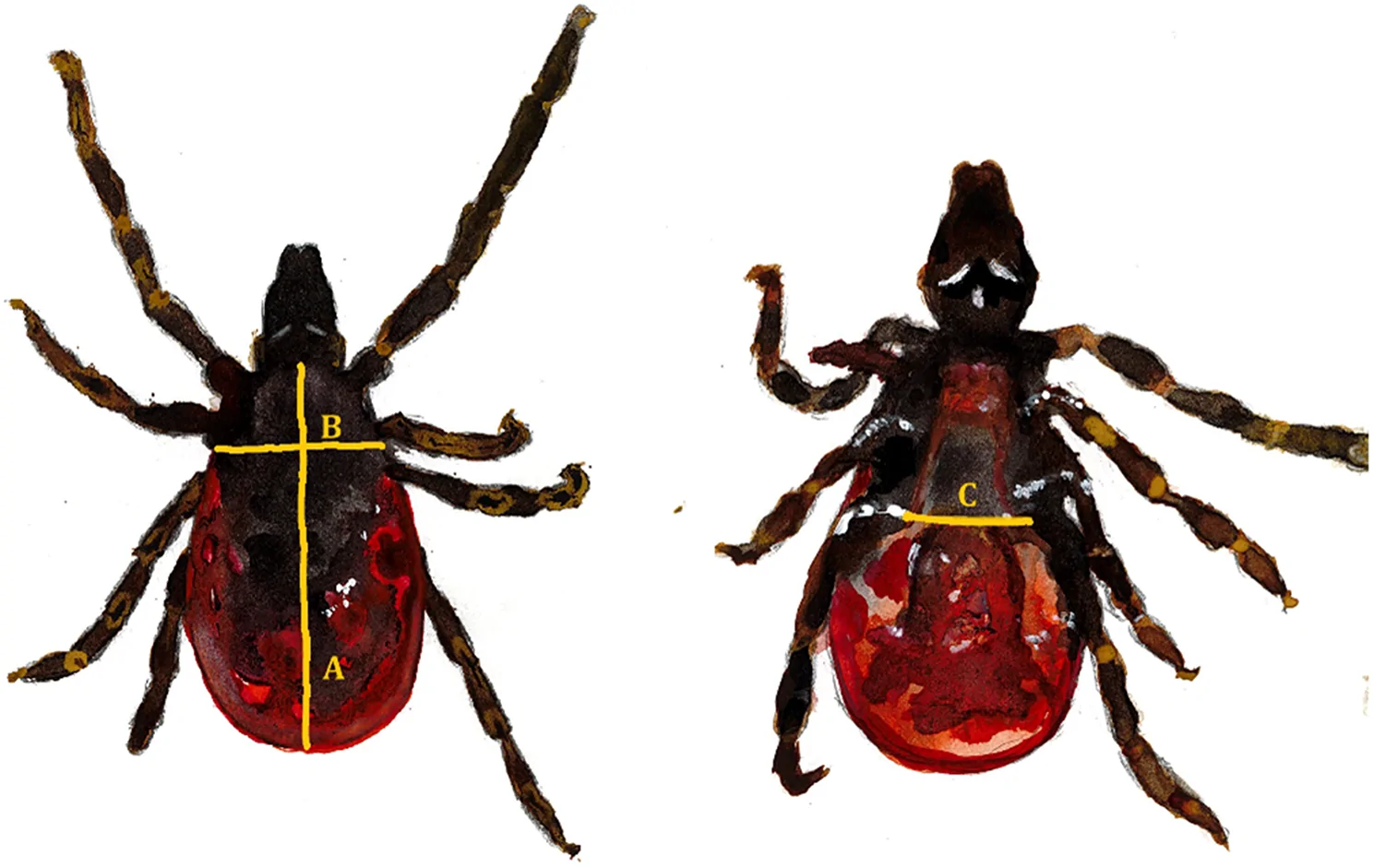
Measurements for feeding duration. Scutal index: idiosoma length (**A**) divided by scutal width (**B**) and coxal gap between the fourth pair of legs (**C**) divided by scutal width (**B**). Original artwork by Emma Lindbom

Ticks collected from wild boar were measured under a light microscope equipped with an ocular micrometer at 40× magnification. For each tick, the scutal index (idiosoma length divided by scutal width) and the coxal index (coxal gap between the fourth pair of legs divided by scutal width) were calculated (Fig. 2). Feeding duration estimates were then derived using the regression equations as previously described [37]. Because the accuracy of these indices varies with engorgement stage, both indices were initially calculated for all ticks [37, 38]. The coxal index was used when feeding duration was estimated to be < 24 h or when the scutal index was unreliable due to specimen condition (e.g. damage or distortion) or in cases of advanced engorgement, where the scutal index equation produced invalid or implausible values due to the rounded body shape. When both indices produced similar values above 24 h, the scutal index was used as the primary estimator.

### Statistical analyses

To assess the associations between *A. phagocytophilum* status (positive, negative) and wild boar life stage (juvenile, adult), gender (male, female) and season (spring, summer, autumn, winter) we used generalized linear models assuming binomial error distribution. The significance of explanatory variables was assessed using likelihood ratio tests. Differences among hunting seasons were analysed with Dunnett’s post hoc test using spring as reference season.

Pathogen transmission can occur in both directions (tick-to-host and host-to-tick). Therefore, two different analyses were performed, one with host qPCR status (positive, negative) as a function of tick status, and one with tick qPCR status as a function of host status.

For the host-level analysis, a generalized linear model was used with host *A. phagocytophilum* status (positive, negative) as the outcome variable, and with the number of *A. phagocytophilum* qPCR positive ticks and total feeding duration summed across all ticks per host as explanatory variables. For the analysis of the odds of *A. phagocytophilum* detection in ticks we used a generalized linear mixed-effects model. In this model, tick qPCR status (positive, negative) was treated as the outcome, and feeding duration and host qPCR status (positive, negative) were included as fixed predictors. Wild boar identity was incorporated as a random factor to account for the dependence of ticks sampled from the same host.

All statistical analyses were conducted in R version 4.4.2 (R Core Team 2024) and Rstudio [39] using packages car [40] and lme4 [41]. GLM models were evaluated using basic diagnostic plots and model check with AIC. For the mixed-effects tick model, residual diagnostics were performed using package DHARMa [42]. A P-value < 0.05 was considered statistically significant. Confidence limits were estimated using Wilson score confidence intervals calculated in OpenEpi [43].

## Results

In total, 88 wild boar were included in the study. Heart tissue was available from all animals (*n* = 88), while spleen (*n* = 87) and urinary bladder samples (*n* = 82) were available from slightly fewer individuals. Attached ticks (*n* = 30) and skin samples from the corresponding attachment sites (*n* = 28) were obtained from 17 wild boar, with some individuals carrying more than one tick. Of the 88 wild boar, 45 (51.1%) were males and 43 (48.9%) were females. Additionally, 47 were juveniles (4–12 months; 53.4%) and 41 were adults (> 12 months; 46.6%). No piglets (< 4 months) were obtained during wild boar sampling. Animals were collected across all seasons, with most samples obtained in winter (*n* = 40), followed by spring (*n* = 21), summer (*n* = 16), and autumn (*n* = 11).

### *Anaplasma phagocytophilum*

**Fig. 3:**
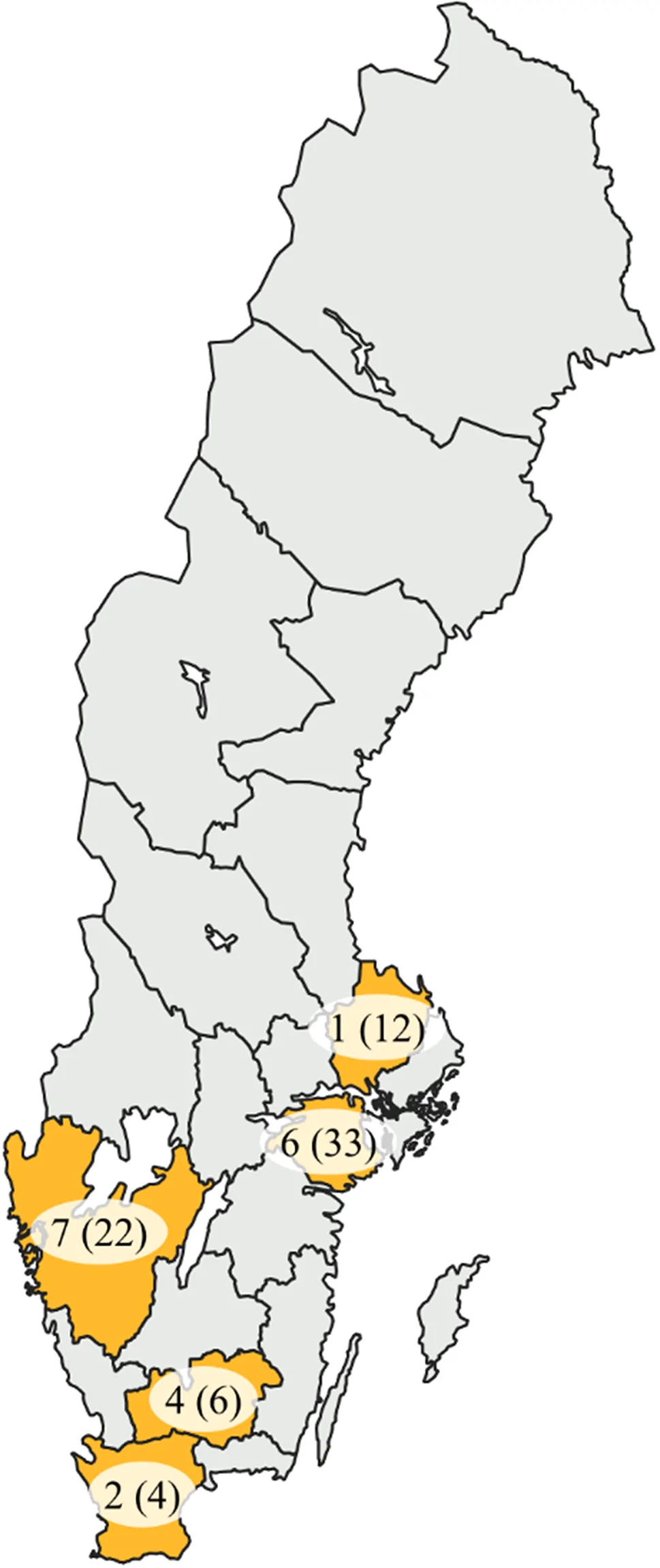
Detection of *Anaplasma phagocytophilum* in wild boar in different counties in southern Sweden. The number of tested animals is shown in parentheses. Västra Götaland: 7 (22), Södermanland: 6 (33), Kronoberg: 4 (6), Skåne: 2 (4), Uppsala: 1 (12). Counts rather than prevalence is presented to avoid misleading impressions in counties with small sample sizes

*Anaplasma phagocytophilum* was detected in 20 of 88 wild boar, corresponding to an overall prevalence of 22.7% (CI95%: 15.2–32.5). Among positive animals (with group sample size, prevalence within group, and 95% CI provided in parentheses), 12 were males (n:45, 26.7%, CI95%: 16.0–41.0) and 8 were females (n:43, 18.6%, CI95%: 9.7–32.6). Regarding age, 15 of the 20 positive wild boar were juveniles (n:47, 31.9%, CI95%: 20.4–46.2) and five were adults (n:41, 12.2%, CI95%: 5.3–25.5) (Table 2). Of the 20 positive animals, one was collected in spring (n:21, 4.8%, CI95%: 0.8–22.7), seven in summer (n:16, 43.8%, CI95%: 23.1–66.8), five in autumn (n:11, 45.5%, CI95%: 21.3–80.0), and seven in winter (n:40, 17.5%, CI95%: 8.7–32.0) (Table 2). *Anaplasma phagocytophilum* was detected throughout the study area (Fig. 3).

**Table 2:** Prevalence of *Anaplasma phagocytophilum* in wild boar. Results are presented by gender, age class, and hunting season (± 95% CI)

Category	Positive for *A. phagocytophilum* *n*	Total *n*	Prevalence within category (CI:95%)^1^
**Gender**			
Male	12	45	26.7% (16.0–41.0)
Female	8	43	18.6% (9.7–32.6)
Total	20	88	22.7% (15.2–32.5)
**Age**			
Piglet: < 4 months	0	0	0%
Juvenile: 4–12 months	15	47	31.9% (20.4–46.2)
Adult: >12 months	5	41	12.2% (5.3–25.5)
Total	20	88	22.7% (15.2–32.5)
**Hunting season**			
Spring [March-May]	1	21	4.8% (0.8–22.7)
Summer [June-August]	7	16	43.8% (23.1–66.8)
Autum [September-November]	5	11	45.5% (21.3–80.0)
Winter [December-February]	7	40	17.5% (8.7–32.0)
Total	20	88	22.7% (15.2–32.5)

^1^ Confidence interval estimated using Wilson score interval

Detection in wild boar of *A. phagocytophilum* was significantly associated with age and hunting season. Juveniles had significantly higher odds of being *A. phagocytophilum* positive compared to adults (OR = 3.19, *P* = 0.048). The odds of *A. phagocytophilum* detection were significantly higher in summer (OR = 14.42, *P* = 0.046) and autumn (OR = 16.26, *P* = 0.048) compared with spring. Gender was not associated with host qPCR status (*P* = 0.57).

In most cases (*n* = 14), *A. phagocytophilum* was detected in a single organ (spleen *n* = 8, heart *n* = 3, urinary bladder *n* = 2, and skin *n* = 1). Of the remaining six animals, three had *A.phagocytophilum* DNA in two organs (spleen and skin *n* = 2; spleen and heart *n* = 1), and three were positive in three organs (spleen, heart and skin *n* = 2; spleen, heart and urinary bladder *n* = 1). Overall, *A. phagocytophilum* was detected in 14 spleen samples (n:87, 16.1%, CI95%: 9.8-25-2), 7 heart samples (n:88, 7.6%, CI95%: 3.9–15.5), 3 urinary bladder samples (n:82, 3.7%, CI95%: 1.3–10.2), 5 skin samples (n:28, 17.6%, CI95%: 7.9–35.6), and 6 ticks (n:30, 20%, CI95%: 9.5–37.3), given a total of 35 positive samples. Of these, ten yielded a sufficiently high-quality sequence for ecotype determination – five spleen, one heart, and four skin samples – all of which were assigned to ecotype I.

### *Borrelia* spp

*Borrelia* spp. DNA was detected in three male wild boar, corresponding to a prevalence of 3.4% (CI95%: 1.2–9.5) among the 88 sampled individuals. All positive animals were juveniles and originated from Kronoberg (*n* = 1) and Sörmland (*n* = 2). The three individuals tested positive in different organs: one in the skin, one in both the urinary bladder and spleen, and one in the heart. Two *Borrelia* detections occurred during the winter hunting season, while one was recorded in summer. Two of the three *Borrelia* spp. positive animals were also co-infected with *A. phagocytophilum*, one sampled in Sörmland and one in Kronoberg.

Of the nine *Borrelia*-positive samples (one spleen, one heart, one urinary bladder, one skin, and five ticks) five (56%) yielded sequences of sufficient quality for species determination: four ticks and one single skin sample. Species assignment identified *B. afzelii* in four of these five samples (three ticks and a skin sample from the tick attachment site): the skin sample and one of the *B. afzelii*-positive ticks originated from the same individual that was also positive for *Borrelia* spp. The remaining tick sample was identified as *B. garinii*, where the corresponding skin sample and wild boar host tested negative for *Borrelia* spp.

Because of the low number of positive cases, no statistical analyses were performed with *Borrelia* spp.

### Ticks and feeding duration

Ticks were collected from 17 wild boar, yielding a total of 30 attached *Ixodes ricinus.* The majority were adult females (*n* = 26), while the remainder were nymphs (*n* = 4). Overall, 10 ticks (n:30, 33.3%, CI95%: 19.2–51.2) were qPCR positive with at least one pathogen. *Anaplasma phagocytophilum* was detected in 6 ticks (n:30, 20%, CI95%: 9.5–37.3) and *Borrelia* spp. in 5 ticks (n:30, 16.7%, CI95%: 7.3–33.6), with coinfection observed in one tick (n:30, 3.3% CI95%: 0.6–16.7).

Feeding duration was successfully estimated for 28 ticks. Both scutal and coxal indices were obtained for 26 ticks, while only one index could be calculated for two ticks due to damage. Feeding duration ranged from 6.4 to 189.0 h (median 65.8 h), with most collected ticks already being late-stage or heavily engorged and only three ticks having attachment times shorter than 24 h. For *A. phagocytophilum*, simultaneous detection in the tick, the skin at the attachment site, and the host was observed in five cases, with feeding durations ranging from 38.1 to 159.9 h. In one case, the tick was positive while the host was negative (64.6 h). Conversely, in five cases the host tested positive, but the tick did not, with feeding durations ranging from 17.6 to 95.5 h. For *Borrelia*, one of the five positive ticks was associated with a positive skin sample, with a feeding duration of 38.1 h. The remaining four ticks were attached to non-infected wild boar, with feeding durations ranging from 45.2 to 82.6 h. Because only three wild boar tested PCR-positive for *Borrelia* spp., the host-level and tick-level analyses were conducted for *A. phagocytophilum* only.

In the host-level model, wild boar *A. phagocytophilum* qPCR status (positive, negative) was used as the response variable, and the number of attached ticks positive for *A. phagocytophilum* and cumulative tick feeding duration were included as explanatory variables. Among the 17 wild boar included in this analysis, 5 carried at least one tick that was positive for *A. phagocytophilum*, while 12 carried only qPCR negative ticks. The odds of *A. phagocytophilum* detection in wild boar were significantly higher in animals infested with a larger number of ticks positive for *A. phagocytophilum* (OR = 23.3, *P* = 0.009), whereas total tick feeding duration showed no significant association with pathogen detection in wild boar (*P* = 0.503).

In the tick-level mixed-effects model, tick qPCR status (positive, negative) was used as the response variable, and host qPCR status (positive, negative) and feeding duration were included as explanatory variables. Host identity was added as a random effect to account for multiple ticks originating from the same animal. Similar to the host-level model, there was no association between feeding duration and *A.phagocytophilum* detection in ticks (*P* = 0.841). However, ticks collected from qPCR positive hosts were more likely to test positive for *A. phagocytophilum* (*P* = 0.013). Estimates for the host-detection effect were very large due to the small sample size and close to total separation, so the emphasis is on the direction and statistical significance rather than the numerical magnitude of the odds ratio.

## Discussion

The prevalence of *A. phagocytophilum* observed in the present study (22.7%) is among the highest reported in Europe. A recent 15-year systematic review of *A. phagocytophilum* in European wild boar reported substantial geographic differences in prevalence across Europe [9]. The highest prevalence values were reported from Slovakia (28.2%) and Poland (20.34%), whereas markedly lower rates were observed in France (2%) and Portugal (3.1%). In addition, several studies from Austria and northern Spain detected no PCR-positive wild boar.

Several factors may contribute to the geographical variation, including methodological differences between studies as well as ecological variation among regions. Methodological factors include differences in sample type, molecular protocols, target genes, PCR approaches, sample size, and geographic coverage, all of which can influence pathogen detection and estimated prevalence. Ecological factors are also likely to influence pathogen circulation. The prevalence of tick-borne pathogens is strongly affected by vector abundance, vector community composition, and host community composition. Areas supporting high densities of *I. ricinus* are generally associated with increased pathogen circulation because they provide greater opportunities for host-vector contact. Differences in tick communities may also contribute to the lower prevalence of *A. phagocytophilum* reported for example in Portugal compared with Sweden. *Ixodes ricinus*, the main vector of *A. phagocytophilum* in Europe, is the dominant tick species in Sweden [13, 14]. In contrast, southern European regions support a considerably more diverse tick fauna. In Portugal, several common tick species coexist, including *Rhipicephalus sanguineus* sensu lato (s.l.), *Dermacentor marginatus*,* Hyalomma marginatum*, and *I. ricinus* [44]. Consequently, the relative contribution of *I. ricinus* in local transmission cycles may differ substantially between regions, potentially contributing to the observed variation in prevalence.

Variation in host community composition may further influence pathogen transmission dynamics. The balance between competent reservoir hosts and non-competent hosts can influence pathogen prevalence in tick populations through amplification or dilution effects. A greater abundance of competent reservoir hosts may increase pathogen circulation and the proportion of infected ticks, whereas a higher abundance of non-competent hosts may reduce transmission by diverting tick blood meals away from competent reservoirs [45]. While the reservoir competence of wild boar has not been established, our findings suggest that they may contribute to the circulation of *A. phagocytophilum*.

Beyond the overall prevalence, the diversity of *A. phagocytophilum* ecotypes maintained by different host species may also influence epidemiological importance and disease risk. Roe deer predominantly sustain ecotype 2, which is generally considered non-zoonotic [15, 46, 47]. In contrast, wild boar have repeatedly been shown to harbour ecotype 1, the ecotype responsible for all confirmed cases of human granulocytic anaplasmosis in Europe [15]. In the present study all ten sequenced wild boar samples were assigned to ecotype I. This distinction highlights important epidemiological differences among host species. While roe deer are regarded as important hosts for maintaining *A. phagocytophilum* in enzootic cycles, their contribution to zoonotic transmission with important implications for human health appears limited. Wild boar may be less infested with ticks but may instead play a more relevant role in the maintenance and circulation of clinically relevant zoonotic strains.

The present study also identified individual-level variation in infection risk. A strong age effect was observed, with juveniles showing approximately threefold higher odds (OR:3.16) of infection than adults. Similar age-related patterns have been reported in roe deer, where younger individuals also exhibit a higher prevalence of *A. phagocytophilum* [48, 49]. Several mechanisms may contribute to this pattern. Younger animals may be more susceptible due to limited acquired immunity resulting from fewer previous exposures to the pathogen.

Younger animals may also have a higher exposure risk due to behavioural differences, movement patterns, habitat use and reduced grooming efficiency. Anatomical differences may also play a role. For example, shorter body dimensions could reduce the distance questing ticks need to move before reaching preferred attachment sites, potentially decreasing the likelihood of removal through grooming. Together, these factors may contribute to the higher infection prevalence observed in juveniles.

Infection rates were lowest in spring, with much higher odds for infection observed during summer (OR:14.42) and autumn (OR:16.26). This pattern is consistent with the seasonal ecology of *I. ricinus*, whose nymphal activity typically follows a bimodal distribution, with a second peak in late summer and early autumn [50], thereby increasing host exposure during these periods. In addition, cumulative exposure to tick bites throughout the tick activity season may increase the probability that wild boar sampled later in the year are already infected, further contributing to the higher prevalence observed during autumn. In contrast to the relatively high prevalence observed for *A. phagocytophilum*, detections of *Borrelia* spp. in wild boar were rare. To the authors’ knowledge, *Borrelia* DNA has previously only been reported in wild boar in Portugal and Italy, with prevalences of 3.3% and 3.0%, respectively [7, 8]. The present study therefore represents the first report of *Borrelia* spp. in wild boar in northern Europe. The prevalence observed (3.4%) closely matches those documented in the two earlier studies.

Species assignment identified *B. afzelii* in four of five samples, consistent with its status as the most commonly detected Lyme borreliosis genospecies in Sweden [34]. The remaining tick sample was identified as *B. garinii*. The *B. afzelii*-positive skin sample and one *B. afzelii*-positive tick originated from the same individual, which also tested positive for *Borrelia* spp. The concurrent detection of *B. afzelii* in the tick, the skin sample from the ticks attachment site, and the host after a feeding duration of only 38.1 h supports active transmission and the establishment of infection. In contrast, the *B. garinii*-positive tick originated from an individual that tested negative for *Borrelia* spp., and whose skin sample from the tick attachment site was also negative despite the prolonged feeding duration (82.6 h), which may suggest that transmission had either not occurred or had not resulted in detectable levels of spirochetes in the skin. However, the number of *Borrelia*-positive ticks was too low to evaluate the relationship between feeding duration and transmission success statistically. Future studies with larger numbers of positive ticks are needed to investigate the significance of feeding duration for *Borrelia* transmission, particularly in relation to different *Borrelia* species.

One important methodological finding of the present study was the value of analysing multiple tissue types. Most previous investigations of *A.phagocytophilum* and *B.burgdorferi* s.l of wild boar have primarily relied either on blood or on spleen tissue [7, 8, 25, 26, 28, 30, 51, 52]. However, experimental infection studies suggest that these tissues may not always be optimal for detecting *Borrelia* spp. In non-human primates, spirochetes have been detected months after infection in several tissues, including the heart and urinary bladder, with the highest bacterial loads observed in bladder tissue [53]. Studies in experimentally infected white-footed mice similarly found *Borrelia* spp. most frequently in urinary bladder (94%), compared with spleen (61%) and blood (13%) [54]. Furthermore, persistent infection of both heart and urinary bladder has been demonstrated in Syrian hamsters, whereas persistence in spleen and liver was shorter and less consistent [55]. Based on these findings, heart and urinary bladder were analysed alongside spleen in the present study to maximise detection sensitivity.

The value of this approach was evident in the results. The three *Borrelia*-positive individuals tested positive in three different organs, one in the skin, one in both the urinary bladder and spleen, and one in the heart. This highlights the risk of underestimating prevalence when only a single tissue is sampled. This pattern is, moreover, broadly consistent with what is known about *B. burgdorferi* s.l. biology. The skin finding is consistent with the local cutaneous replication that occurs at the site of tick attachment during initial infection, a feature common to all *B. burgdorferi* s.l. genospecies [56], while the urinary bladder and heart findings are consistent with the higher bacterial loads and persistent infection reported for these organs in the experimental studies described above. Spleen, by contrast, is not an organ where this spirochete typically establishes a persistent infection, and bacterial loads there have similarly been reported to be lower and less consistent than in bladder or heart, yet its role as a phagocytizing, blood-filtering organ makes the presence of circulating spirochetes biologically plausible, particularly during the brief bacteraemic phase that precedes dissemination to other tissues.

The inclusion of multiple tissue types in the analysis may also increase the detection probability for *A. phagocytophilum*, although for somewhat different biological reasons than for *Borrelia* spp. In general, blood or spleen samples are considered suitable materials for determining the prevalence of this obligate intracellular bacterium. In humans, the pathogen can remain detectable in the bloodstream for up to 28 days, and persistent infections have been documented in several animal species, including sheep, horses, dogs, cattle, and red deer [57]. Consequently, PCR-based diagnostics are routinely used for detecting *Anaplasma* spp. infections in both humans and domestic animals [58, 59]. However, when bacterial loads are very low, infected neutrophils may be unevenly distributed within host tissues, which can reduce the probability of detection when only a single tissue type is analysed. This pattern was also observed in a study of paired liver and spleen samples from 59 wild boar. Of the 12 individuals that tested positive, only one contained *A. phagocytophilum* DNA in both organs, whereas all other positives were detected in just one tissue [60]. The value of a multi-tissue approach was similarly evident in the present study. If only spleen samples had been analysed, 14 positive cases would have been detected, compared with the 20 cases identified when multiple tissues were included. Two of these additional positives were detected exclusively in the urinary bladder. Although this finding may initially appear unexpected, it is most likely explained by the incidental presence of infected leukocytes within the sampled tissue rather than by organ-specific tropism of *A. phagocytophilum*. Because *A. phagocytophilum* resides within circulating leukocytes, any vascularised tissue may contain infected cells, and when bacterial loads are low, detection in a particular tissue may depend partly on chance.

In addition, a notable limitation of the study was the low sequencing success among qPCR-positive samples. Although both *A. phagocytophilum* and *Borrelia* spp. were detected in several samples, only a subset yielded sequences of sufficient quality for downstream analyses. Similar discrepancies between pathogen detection and successful sequencing have been reported previously. In a study of *A. phagocytophilum*, only 206 of 810 qPCR positive tick samples yielded sequences suitable for ecotype determination, with sequencing success decreasing markedly at lower pathogen copy numbers [61]. The low sequencing success observed may therefore reflect limited quantities of amplifiable template DNA, which were sufficient for qPCR detection but insufficient for the additional amplification and sequencing steps required for species or ecotype determination.

Beyond the pathogen detections and characterization, the occurrence of attached ticks provides additional evidence of frequent host-vector contact in wild boar. One commonly stated reason for the limited consideration of wild boar in the epidemiology of tick-borne diseases is the perception that this species carries relatively few ticks. However, a notable number of ticks were detected on wild boar in the present study despite the absence of systematic or exhaustive tick searches. Because tick collection relied on voluntary hunters and was conducted under varying field conditions, it is likely that some attached ticks were not detected or collected. Consequently, the recorded tick burden should be considered conservative and likely represents an underestimation of the true exposure.

Combined with the relatively high prevalence of tick-borne pathogens detected in wild boar, these findings suggest frequent contact with ticks and highlight that wild boar may be more involved in tick-borne disease ecology than is often recognized.

A tentative positive association between infection in ticks and infection in wild boar was observed. Due to the small number of ticks included the result needs to be interpreted with caution. Furthermore, because both tick and host infection were measured at a single time point, the direction of transmission cannot be determined; an infected tick may have acquired the pathogen from the host, the host may already have been infected prior to tick attachment, or both may have been infected independently. Despite these limitations, the observed correspondence between host and tick infection is broadly consistent with wild boar being involved in the epidemiology of *A. phagocytophilum*, at least as exposed and potentially infectious hosts within the local transmission system.

## Conclusions

This study provides updated information on the occurrence of *A. phagocytophilum* and *Borrelia* spp. in Swedish wild boar and their attached ticks. The relatively high prevalence of *A. phagocytophilum* adds to growing evidence that this pathogen occurs regularly in wild boar populations. Because wild boar can carry *A. phagocytophilum* lineages belonging to ecotype I, the ecotype most often associated with human-pathogenic strains in Europe, this host species is relevant when assessing local transmission dynamics. We also report the first detection of *Borrelia* spp. in wild boar in northern Europe, but the low prevalence observed suggests that wild boar likely play only a limited role in the maintenance of *Borrelia* spp. in natural transmission cycles. The use of multiple tissue types increased detection sensitivity for low-level infections, and matching host–tick samples provided additional context for interpreting infection patterns. Larger and more complete datasets, ideally including systematic tick collection and consistent sampling of key organs, will be important for clarifying the role of wild boar in the circulation of tick-borne pathogens in northern Europe.

## Data Availability

The datasets used and/or analysed during the current study are available from the corresponding author on reasonable request.

## Declarations

### Prior publication

Data have not been published previously.
